# 1392. An observational study to test efficacy of oral fluconazole in pediatric cases of Cutaneous Leishmaniasis

**DOI:** 10.1093/ofid/ofad500.1229

**Published:** 2023-11-27

**Authors:** Debdeep Mitra

**Affiliations:** Command Hospital Air Force Bangalore, Bangalore, Karnataka, India

## Abstract

**Background:**

Cutaneous Leishmaniasis(CL) is the commonest form of Leishmaniasis with a worldwide incidence of about 0.7 to 1.2 million cases per year and 90% of these cases are in Asia, the Middle East, South America, and North Africa. Pentavalent antimony medications are still the most effective treatments for leishmaniasis, however, due of the severe side effects and the need for parenteral administration, other therapies, such as oral medicines for cutaneous leishmaniasis, have been sought. Given the extended duration of the illness and the disfiguring impact of unhealed skin lesions, a well-tolerated oral medication would be beneficial. In vitro, certain azole antifungal medications exhibit cidal action against leishmania. Fluconazole, a triazole antifungal drug available for oral administration, has clinical efficacy against leishmania but very limited data is available to support this.

**Methods:**

To test the efficacy of oral fluconazole in CL in children we treated 14 Indian patients of age less than 12 years with once daily doses of fluconazole for four weeks. Patients less than 12 years old with clinically and smear positive confirmed cases were included in the study. Immuno-compromised patients were excluded.

**Results:**

In the majority of patients, a parasitological smear was used to confirm the diagnosis, and in a few cases, histological testing was used. 10 patients were male, and mean values (SE:Standard error) at study entry were: age, 7(2) years; weight, 20 (3) kg; duration of illness, 2·4 (0·4) months. All children were prescribed fluconazole dispersible tablet at the dose of 3-5 mg/kg body weight to adjust doses in multiple of 50 mg of dispersible tablet daily. No drug discontinuation or any case drop out was noted as the drug was well tolerated and there was good noticeable clinical response. Intermittent nausea and occasional diarrhea was the most common side effect (7% of cases). Clinical cure was noted with complete clearance of skin lesions in all 14 cases and no appearance of any new lesions was observed over next 3 months. The average treatment response time was 43 days

**Conclusion:**

There are very limited studies on the use of fluconazole in pediatric CL in endemic areas and this study suggests a very safe and effective treatment option.

**Disclosures:**

**All Authors**: No reported disclosures

